# Leadership Power Perceptions of Soccer Coaches and Soccer Players According to Their Education

**DOI:** 10.2478/v10078-012-0073-x

**Published:** 2012-10-23

**Authors:** Erkut Konter

**Affiliations:** 1 Buca Educational Faculty, Dokuz Eylül University, İzmir, TURKEY.

**Keywords:** perception of leadership power, soccer coaches, soccer players, education

## Abstract

The purpose of this study was to analyze the leadership power perceptions of soccer coaches and soccer players according to their educational levels. Data were collected from 165 male soccer coaches and 870 male soccer players. Adapted versions of the “Power in Soccer Questionnaire-Other”, the “Power in Soccer Questionnaire-Self” and an “information form” were used for data collection, and collected data were analyzed by the Kruskal-Wallis and the Mann-Whitney Tests. Analysis of the Power in Soccer Questionnaire-Other revealed significant differences between soccer players’ level of education and their perception of Coercive Power (p<.003), and no significant differences related to Referent Power, Legitimate Power and Expert Power. Analysis of the Power in Soccer Questionnaire-Self also revealed the only significant difference between coaches’ level of education and their perception of Legitimate Power (p<.001), and no significant differences with regard to others. Different perception of leadership powers between coaches and players might create communication and performance problems in soccer.

## Introduction

Sport leadership has been a very intriguing area for practitioners and researchers. Not only have sport psychologists attempted to better understand the role of psychological factors in sport in general but also they have begun to examine the role of leadership and coaching in particular sport disciplines (Chelladurai, 1990; Gould, 2008).

Leaders’ influence and followers’ compliance have frequently been studied in social and organizational psychology within a theoretical framework known as the bases of social power (French and Raven, 1959; Frost and Stahelski, 1988). French and Raven (1959) identified five sources of interpersonal power that are Reward Power, Coercive Power, Referent Power, Legitimate Power and Expert Power in leadership.

Wann et al. (2000) adapted these interpersonal powers to sport settings showing the psychometrically sound validity and reliability results of the five-factor model in North America. Konter (2009) recently adapted these scales to the Turkish language using amateur and professional soccer players and coaches. Despite the importance of the subject in sport psychology, there are only few research related to leadership power in sport (Wann, et al., 2000; Konter, 2009) and soccer in particular.

People in general, coaches, sport officials, players, and even spectators in particular, possess power to the extent that they have the ability to influence or change the attitudes or behaviors of others (French and Raven, 1959) in a socio-cultural environment. French and Raven (1959) emphasized five interpersonal or social powers: Reward, Coercive, Legitimate, Expert and Referent Powers.

Reward power involves the ability to reward others such as verbal praise, positive body language, and more playing time. Meanwhile Coercive Power concerns the ability to control access to one or more punishments for example verbal reprimands, negative gesture, giving less playing time, making players run laps or perform sit ups and push-ups. Legitimate Power involves the ability to use one’s position and authority within the organization, group or team, for example, being an authority figure, possessing official status, ownership of the organization, being the head coach etc. Expert Power is derived from perception that one is knowledgeable, skillful, or talented in a specific domain. For example, Expert Power consists of being a former star in that sport, having specific education and experience, awarded many titles or medals. Finally, Referent Power involves the ability to be liked and respected by the group members. For example, athletes respect and admire their coaches, and follow their decisions (French and Raven, 1959; Konter, 2009; Wann et al., 2000).

Number of studies claim that coaches’ and athletes’ perception of power can affect satisfaction, ability, positive assessment, team cohesion, imagery, coping with stress and control of competitive anxiety, success and performance indicated that coaching, in general and specifically, involves developing the sporting ability of athletes (Expert Power). Some research also showed that; a) relationship between coaches and athletes is a dynamic process, changing over time and conditions, effecting the thoughts, emotions and behaviors of both sides, b) coaches prefer more authoritarian style (Legitimate Power and Coercive Power) than athletes, c) coaches caring thoughts and emotions of their athletes (Referent Power), develop better relationship with them (Chelladurai et al., 1989; Home and Carron, 1985; Reimer and Chelladurai, 1995; Garland and Barry, 1990; Gordon, 1988; Laughlin and Laughlin, 1994; Maby, 1997; Konter, 2005; Lyle, 2002; Jowett and Poczwadowski, 2007; Salminen and Lukkonen, 1996; Summers and Russel, 1991).

Gould et al. (1990) revealed that seminars and text books have a minimal effect in informational resources of coaches (Expert Power). The research indicated that factors leading to success for coaches consist in learning from their own experience, applying their own style, adapting the changing conditions and monitoring other successful coaches (Expert and Referent Power). Bloom (1997) argued that athletes working with coaches, who have hard work quality (Expert Power) and better communication skills (Referent Power), are more satisfied, more successful, have better developed skills, more chance of self realization and self actualization. According to Bloom (1997), successful coaches generally follow update knowledge in their specific field, do hard work to continually develop themselves, share and exchange information with their colleagues, participate in seminars, courses and conferences, read books and use related technological documents in their sports, and stay constantly in coaching experience (Expert Power). Gould et al. (2006; 2007) studied characteristics of high school football coaches who were recognized for developing character and positive personal characteristics in their players (Expert and Referent Powers). They did in-depth phone interviews with 10 finalists for the National Football League Charities “Coach of the Year Program”. Ten of coaches’ former players were also interviewed. They indicated that, while these coaches highly motivated to win, they made the personal development of their players a top priority (Expert and Referent Powers). In addition, these coaches had well thought-out coaching philosophies and clear expectations relative to rules (Expert Power), player behaviors, and team needs (Referent Power). Gould et al. (2006; 2007) concluded that, while common themes and patterns were evident across coaches, each coach focused on a relatively small number of individual specific key principles such as having discipline (Legitimate and Coercive Powers), working hard, being totally prepared (Expert Power) and respecting and putting one’s family before other needs (Referent Power).

It can be concluded that leadership styles (relationship orientation versus task orientation etc.) are associated with age, skill and education levels. For example sport participants need a) low Expert Power (task orientation) and high Referent Power (relationship orientation) at primary and secondary school levels, b) low-medium Expert Power and high-medium Referent Power at high school level, c) high-medium Expert Power and low-medium Referent Power at university level (Chelladurai and Carron, 1978; Leith, 2003). Chelladurai and Carron (1978) found that participants who have less maturity in sport skill, demand more Referent Power (relationship orientation) whereas, more mature participants in their skills need it less. In addition, Chelladurai and Carron (1983) stated that athletes look for more social support (Referent Power) with the increase of age, maturity and experience, while training and instruction (Expert Power) drop in high school (ages 14–15 to 18–19), but increase again during the years of higher education (ages 18–24). Home and Carron (1985) showed in their research that coaches perceive themselves differently than their athletes. They indicated that coaches assess themselves much more positively (for example training and instruction, democratic behavior, positive feedback and social support) than their athletes in all leadership factors (except autocratic behavior).

The forms of the PSQ (PSQ-O and PSQ-S) related to sport education would be of value to sport psychology because they may provide information about teams’, players’ and coaches’ perception, cognition, behavior, communication, leadership, satisfaction, performance and other factors involving the socio-psychological nature of soccer. In general, globalization of sports and education make international and cross cultural nature of management, education and leadership more important in sport organizations (Allcorn, 1997; Maças et al., 2007). Particularly, soccer is the most popular sport in the world and it becomes a more international and cross cultural sport activity involving millions of people and multi billion dollar business (Allcorn, 1997; Maças et al., 2007). Therefore, leadership and education of players, coaches, teams, administrators and other related supporters have become of great importance in order to obtain the desired results, success, performance and satisfaction in sport organizations. Therefore, the purpose of this study was to analyze the leadership power perceptions of soccer coaches and soccer players (Coercive, Expert, Legitimate, and Referent Powers) according to their educational levels (primary school, high school and university level of education).

## Material and Methods

### Participants

Data were collected from 165 male soccer coaches (71 Technical Directors and A License, 46 B License, 48 Amateur License) and 870 male soccer players (173 professionals and 697 amateurs) in Turkey. The coaches had a mean age of 40.24 years ± 8.40 and had been coaching for an average of 8.56 ± 6.75 years. The players had a mean age of 18.40 ± 4.00 years and had been playing soccer for an average of 6.00 ± 4.15 years with a license.

### Instruments

Wann et al. (2000) pioneered using French and Raven’s five interpersonal powers construct in sports and developed the Power in Sport Questionnaire-Other (PSQ-O for athletes) and Power in Sport Questionnaire-Self (for coaches). Konter (2009) adapted the PSQ forms for Turkish athletes and coaches. Turkish version of the PSQ-O has a total of 10 items and the PSQ-S has 11 items with four factors including Coercive, Referent, Legitimate and Expert Powers. Analysis of the PSQ-O and the PSQ-S for Turkey revealed that the subscales were acceptable for research purposes and were internally consistent ranging from 0.58 to 0.70 for the PSQ-O and from 0.62 to 0.84 for the PSQ-S (Reynolds et al., 2006). The PSQ-O and the PSQ-S are Likert Type-Scale formats and responses to each item range from 1 (this is very untrue) to 9 (this is very true).

An item example of each factor of the PSQ-O and the PSQ-S are as follows: on the PSQ-O, an item (item 2) on the coercive power factor read “I do what this person/these persons ask and I abide by their decisions because they have the ability to punish me”. In contrast, on the PSQ-S, this item read, “Others do what I ask and abide by my decisions because I have the ability to punish them”. On the PSQ-O, an item (item 3) on the referent power factor read “I do what this person/these persons ask and I abide by their decisions because I like them”. In contrast, on the PSQ-S, this item read, “Others do what I ask and abide by my decisions because they like me”. On the PSQ-O, an item (item 4) on the legitimate power factor read “I do what this person/these persons ask and I abide by their decisions because they are in charge in this sport”. In contrast, on the PSQ-S, this item read, “Others do what I ask and abide my decisions because I am in charge in this sport”. On the PSQ-O, an item (item 5) on the expert power factor read “I do what this person/these persons ask and I abide by their decisions because they have a great deal of knowledge about this sport”. In contrast, on the PSQ-S, this item read, “Others do what I ask and abide by my decisions because I have a great deal of knowledge about this sport”.

### Procedures and Data Collection

Adapted Turkish version of the PSQ-O, the PSQ-S (questionnaires) and an information form related to demographic variables including the educational level of soccer players and coaches were applied for data collection. Head coaches for soccer clubs were contacted and the nature of the research project was explained. The coaches were informed that the research involved coaches’ and athletes’ perceptions about leadership powers in soccer. After coaches and soccer players consented to participate in the research, a meeting time and place for survey sessions were determined.

During the testing session, players were briefly given information about the research project and they were encouraged to answer the questionnaire honestly. They were also asked not to put their names on the forms and informed that their answers would only be used for research purposes and kept confidential. The PSQ forms with brief instructions were then administered to players (the PSQ-O) and coaches (the PSQ-S). Both PSQ forms also had some demographic questions to collect information about participants’ age, gender, sport and years of experience and educational level. Completion of the each PSQ form required approximately 10–15 minutes.

### Analysis of Data

The Kruskal-Wallis and the Mann-Whitney Tests were applied for the both collected data set (the PSQ-O for soccer players’ data and the PSQ-S for soccer coaches’ data). Nonparametric tests of the Kruskal-Wallis and the Mann-Whitney were used for the analyses, since the both data set are not normal distributed and the one group of the independent variable has a relatively low participant number (24 university level soccer players related to the PSQ-O form and 18 primary school level coaches related to the PSQ-S form). SPSS 15.0 program was used for the data analyses. Comparisons were made between the four dependent (perceptions about Coercive, Legitimate, Expert and Referent Powers), and the three independent variables (primary school, high school and university education) of players and coaches. Results of the analyzed date are presented below (Table 1 and Table 2). Significant differences were determined by p < 0.05.

## Results

### Results of PSQ-O (Soccer Players’ Form)

The Kruskal-Wallis Test results of the PSQ-O for soccer players according to their educational levels are presented in Table 1.

The Kruskal-Wallis Test of the PSQ-O revealed significant differences between the soccer players’ level of education and their perception of Coercive Power [χ^2^ (2) = 11.68, p < 0.003]. However, no significant differences were found between soccer players’ level of education and their perception of Referent Power, Legitimate Power and Expert Power (p >.05). Mean rank analyses showed that soccer players with primary school education have the highest value (Mean Rank = 460.46), and this value drops with the high school education (Mean Rank = 401.45) then increases with the university education again (Mean Rank = 441.29). Comparative analyses of soccer players using the Mann-Whitney Test revealed significant difference between primary school education and high school education (p <.001). The results indicated that perception of Coercive Power drops from primary school to high school, and then increases from high school to university education.

### Results of PSQ-S (Soccer Coaches’ Form)

The Kruskal-Wallis Test results of the PSQ-S for soccer coaches according to their educational level are presented in Table 2.

The PSQ-S test revealed the only significant difference between the coaches’ level of education and their perception of Legitimate Power [χ^2^ (2) = 13.58, p < 0.001], and no significant differences between the coaches’ level of education and their perception of Coercive, Referent and Expert Powers (p > 0.05). Mean Rank Analyses showed that; coaches with primary school education (Mean Rank =106.58) have higher perception of Legitimate Power than the coaches with high school education (Mean Rank = 93.41) and university education (Mean Rank = 70.14). The Mann-Whitney Test revealed a significant difference between soccer coaches with primary school education and university education (p < 0.003). In addition, analyses showed significant differences between high school and with university education of soccer coaches (p < 0.004).

Results indicated that the more education coaches have the less Legitimate Power they perceive.

## Discussion

Results, as a whole, indicate that there are similarities and differences related to power perception of soccer coaches and players. There are similarities related to Expert Power and Referent Power, while differences exist in regard to Coercive Power and Legitimate Power perceptions of soccer coaches and players. This result indicates that soccer coaches and players, to the degree, perceive leadership powers differently. These different power perceptions of coaches and players might create some problems of communication, success and performance.

Results of the study showed that there are meaningful differences between educational level of soccer players and their perceptions of Coercive Power. Soccer players with primary school diplomas have higher Coercive Power than soccer players with high school diplomas. These results do not support the findings of previous research (Chelladurai and Carron, 1978).

It might be suggested that the more educated players are, the more effectively they can solve their problems, feel more independent, and have less need for Coercive Power. Rather soccer players with a higher educational level, prefer and like not being forced or pushed by coercive leadership. Therefore, coaches should know that players would be in a different need of Coercive Power related to their educational level. Coaches particularly, take into consideration that the less educational level of players, the more they need Coercive Power. In this case, leadership style of coaches might change from democratic or liberal to authoritarian.

Similarly, the higher educational level of players, the less they perceive Referent Power with their coaches. This might be as a result of educational effect that makes players perceive more independent. Players could also perceive more work ethics and do their jobs more professionally, take more responsibility and act more mature with the effect of education rather than just identifying themselves (Referent Power) with their coaches. Chelladurai and Carron (1978) as well as Leith (2003) indicated that relationship orientation (Referent Power) of players drops with the increase of their educational level from primary school to university and conversely, task orientation (Expert Power) increases with the level of education from primary school to university. But, with the drop of Referent Power perception of players as a result of higher educational level can affect the factors related to team cohesiveness and group dynamics (for example, social cohesion and task cohesion). The more educational level players have, the more they could feel responsibility for their roles and follow individual development related to the tasks instead of simply liking and respecting their coaches.

Applying Coercive Power for educated players can set coaches for failure. Similarly, using liberal or democratic approach for less educated players may yield unsuccessful results for coaches. Therefore, some coaches might prefer the authoritarian style with father like attitude to their less educated players. The results of the present study support these contentions related to coaches’ perception of Coercive Power.

Analyses of the PSQ-S (coaches’ form) only revealed a significant difference between coaches’ level of education and their perception of the Legitimate Power. The analyses yielded that coaches with primary school education have higher perception of Legitimate Power than the coaches with high school and university education. The results indicated that the more education coaches have the less Legitimate Power they perceive.

It can be inferred from the present study that the level of education might affect coaches’ perception of the Legitimate Power more than the Expert Power. The Analyses of the PSQ-S also revealed that the education level of coaches is not a meaningful factor regarding Coercive Power, Referent Power and Expert Power. This result appears to be contradictory in regard to the impact of education itself on Expert Power. This contradictory result might be related to the other informal educational activities of coaches rather than just their formal school education. In addition, perception of Coercive Power, Referent Power and Expert Power might be important at all education levels for coaches in soccer. Therefore, future research should consider the informal education of coaches and players as indicated by Bloom (1997) and Gould et al. (1990).

The results of the present study also yielded that soccer coaches and players perceive the Coercive Power and the Legitimate Power differently, whereas they perceive Referent Power and Expert Power similarly. This result could indicate that perception of Referent Power and Expert Power for soccer coaches and players are important at all levels of the formal education system. This result partly supports previous research (Home and Carron, 1985).

Above contradictory results of power perception might create problems of communication, assessment, satisfaction including success and performance between soccer coaches and players. Thus, soccer clubs look for assistance from sport psychologists to improve the relationship between coaches and players, and improve their performance.

Perception of leadership powers including motivational orientations and cognitive structure might be related to personality traits of soccer coaches and players apart from their educational level (Chelladurai and Carron, 1981; Erle, 1981; Vallerand and Ratelle, 2002). Therefore, it is difficult to make generalizations and comment previous research findings since there have been limited research related to the coaches’ and players’ perception of leadership power including individual and situational variables in soccer. Future research might also consider the level of education obtained from coaching courses. In addition, new projects should concentrate on life skill development related to perception of leadership power and education in soccer.

There is obviously more research needed to have definite conclusions regarding the leadership power perception of soccer coaches and players. Scientists should not only consider the soccer coaches’ and players’ level of formal education at schools, but also the level of coaching education, skill, different league status, professional and amateur participation, and other educational efforts, such as reading books, participating in seminars, training and diagnostic courses and conferences, and following technological development in soccer equipment.

## Figures and Tables

**Table 1:** Soccer players’ leadership power perception according to their educational levels.

PSQ	N	M	SD	SE	Mean	df	χ^2^	p	Sig.

EDU.					Rank				
COERCIVE POWER/EDUCATION						2	11.68	.003	YES
1) Prim.	462	9.18	4.96	.29	460.46				Between 1 and 2
2) High	381	8.00	4.69	.24	401.45				
3) Univ.	24	8.71	4.40	.90	441.29				
TOTAL	870	8.65	4.86	.16					
REFERENT POWER/EDUCATION						2	4.25	.119	NO
1) Prim.	462	19.00	5.83	.27	420.15				
2) High	381	19.94	4.97	.25	453.23				
3) Univ.	24	19.08	4.32	.88	395.31				
TOTAL	870	19.41	5.44	.18					
LEGİTİMATE POWER/EDUCATION						2	.50	.779	NO
1) Prim.	462	13.85	4.05	.19	434.42				
2) High	381	14.04	3.56	.18	431.33				
3) Univ.	24	14.79	2.83	.58	468.23				
TOTAL	870	13.96	3.81	.13					
EXPERT POWER/EDUCATION						2	3.01	.222	NO
1) Prim.	462	22.52	4.29	.20	444.35				
2) High	381	22.13	4.49	.23	425.73				
3) Univ.	24	21.37	4.26	.87	366.06				
TOTAL	870	22.32	4.38	.15					

**Table 2:** *Soccer coaches’ leadership power perception according to their educational levels*.

PSQ	N	M	SD	SE	Mean	df	χ^2^	p	Sig.

EDU.					Rank				
COERCIVE POWER/EDUCATION						2	2.52	.28	NO
1) Prim.	18	12.28	5.92	1.4	82.69				
2) High	63	12.95	5.39	.68	90.24				
3) Univ.	84	11.34	5.13	.56	77.64				
TOTAL	165	12.06	5.33	.41					
REFERENT POWER/EDUCATION						2	3.04	.22	NO
1) Prim.	18	19.28	5.58	1.3	76.33				
2) High	63	20.88	4.75	.60	91.19				
3) Univ.	84	19.87	4.30	.47	78.29				
TOTAL	165	20.19	4.63	.36					
LEGİTİMATE POWER/EDUCATION						2	13.58	.001	YES
1) Prim.	18	14.39	2.99	.70	106.58				Between 1 and 3, 2 and 3
2) High	63	13.05	4.30	.54	93.41				
3) Univ.	84	11.44	3.83	.42	70.14				
TOTAL	165	12.38	4.05	.31					
EXPERT POWER/EDUCATION						2	.23	.89	NO
1) Prim.	18	21.28	5.11	1.2	85.25				
2) High	63	21.50	4.47	.56	84.66				
3) Univ.	84	21.23	4.26	.46	81.27				
TOTAL	165	21.34	4.41	.34					
